# The complete chloroplast genome of the endangered species *Cephalotaxus hainanensis* (Cephalotaxaceae)

**DOI:** 10.1080/23802359.2018.1532838

**Published:** 2018-10-26

**Authors:** Shujun Chen, Shuoxin Zhang, Jian Huang

**Affiliations:** a College of Forestry, Northwest A&F University, Yangling, China;; b Qinling National Forest Ecosystem Research Station, Ningshan, China;; c Key Comprehensive Laboratory of Forestry, Shaanxi Province, Yangling, China

**Keywords:** *Cephalotaxus hainanensis*, Illumina sequencing, chloroplast genome, phylogenetic analysis

## Abstract

*Cephalotaxus hainanensis* is an endangered tree endemic to China with high medicinal values. In this study, the complete chloroplast genome of *C. hainanensis* was *de novo* assembled using Illumina high-throughput sequencing technology. The whole chloroplast genome was 136,877 bp long with an asymmetric base composition (32.6% A, 17.6% C, 17.4% G and 32.4% T). The genome annotation predicted a total of 114 genes, including 82 protein-coding genes, 28 tRNA genes, and four rRNA genes. The neighbour-joining phylogenetic analysis based on 41 complete chloroplast genome sequences showed that *C. hainanensis* was more closely related to the congeneric *C. sinensis*. The assembled chloroplast genome of *C. hainanensis* will provide useful genomic data both for the phylogenetic research of *Cephalotaxus* and the conservation of this endangered species.

*Cephalotaxus hainanensis*, an evergreen medicinal plant in the family Cephalotaxaceae, is distributed in the narrow range of the western Guangdong, Hainan, southeastern and western Guanxi, western Yunnan, and even southeastern Tibet, China (Yang and Liao 2013; Qiao et al. 2014). This plant is suggested to have antileukemia, antidiabetic, anticancer and antitumor activities and is widely used as a herbal remedy in China (Pan et al. 2010; Liu et al. 2016). However, due to logging and more recently exploitation of bark and leaves which contain the valuable medicinal extracts, cephalotaxine and harringtonine, the number of the wild *C. hainanensis* has decreased sharply (Qiao et al. 2014). At present, this species is listed in IUCN Red List of Threatened Species regarded as ‘endangered’ (Yang and Liao 2013). To facilitate its phylogenetic analysis and conservation genetics of this endangered species and sustainable utilization, we assembled its complete chloroplast genome by Illumina sequencing technology in this study. The chloroplast genome sequence was submitted to GenBank (accession number MH311692).

Fresh leaves were collected from a single individual of *C. hainanensis* in Lushan Botanical Garden (Jiujiang, Jiangxi Province, China). The genomic DNA was extracted using the DNeasy Plant Mini Kit (QIAGEN,  Mountain View, CA). High-throughput sequencing was conducted on the Illumina HiSeq platform. A total of 43,729,560 of 150 bp raw paired reads were generated, quality-trimmed with CLC Genomics Workbench v10 (CLC Bio, Aarhus, Denmark), and subjected to the assembly of chloroplast genome with MITObim v1.9 (Hahn et al. 2013). The chloroplast genome of *Cephalotaxus oliveri* (KC136217) (Yi et al. 2013) was selected as the initial references. Genome annotation was conducted in GENEIOUS R11 (Biomatters Ltd., Auckland, New Zealand) by aligning with those of phylogenetically related species. A physical map was drawn with the web-based tool OGDRAW (Lohse et al. 2013).

The chloroplast genome of *C. hainanensis* was successfully assembled with an average coverage of 152× and was determined to be 136,877 bp in length. As commonly found in chloroplast genomes of other higher plants, the base composition was asymmetric (32.6% A, 17.6% C, 17.4% G and 32.4% T) with an overall A + T content of 65.0%. It encodes a set of 114 unique genes, including 82 protein coding, 28 tRNA and four rRNA genes. Almost all genes occurred in a single copy with the exception of *trnQ-UUG* being duplicated. Among these genes, 14 genes harboured a single intron (*atpF, ndhA, ndhB, petB, petD, rpl2, rpl16, rpoC1, rps12, rps16, trnA-UGC, trnG-UCC, trnI-GAU* and *trnK-UUU*) and 1 genes (*ycf3*) harboured two introns.

To investigate the phylogenetic status of *C. hainanensis*, a neighbour-joining phylogeny was reconstructed from the complete chloroplast genomes of 41 species within the order Cupressales using the concatenated sequences of chloroplast protein-coding genes with MEGA6 (Tamura et al. 2013) performed with 500 replicates (Figure 1). The phylogenetic analysis indicated that *C. hainanensis* was more closely related to the congeneric *Cephalotaxus sinensis* (Duan et al. 2018). The complete chloroplast genome of *C. hainanensis* will supply useful genetic information for population genomic studies, and conservation management of this endangered species.

**Figure 1.**
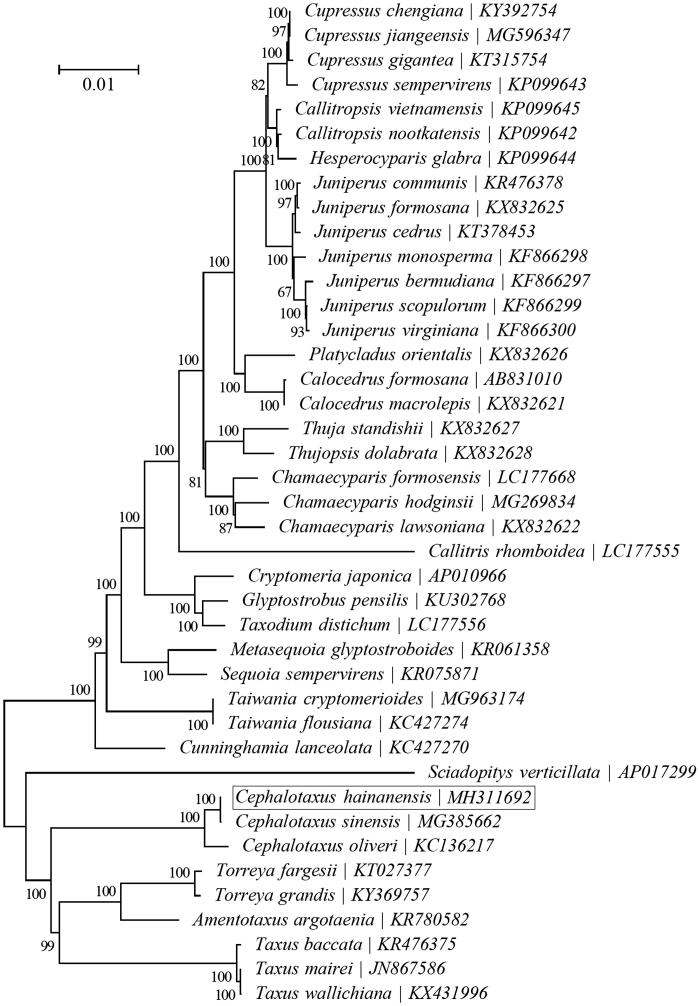
Phylogenetic relationships of 41 species based on the neighbour-joining analysis of chloroplast protein-coding genes. The bootstrap values were based on 500 replicates, and are shown next to the branches.
